# Re-infection outcomes following one- and two-stage surgical revision of infected hip prosthesis in unselected patients: protocol for a systematic review and an individual participant data meta-analysis

**DOI:** 10.1186/s13643-015-0044-0

**Published:** 2015-04-25

**Authors:** Setor K Kunutsor, Michael R Whitehouse, Jason Webb, Andrew Toms, Ian Stockley, Adrian Taylor, Stephen Jones, Matthew Wilson, Ben Burston, Tim Board, John-Paul Whittaker, Ashley W Blom, Andrew D Beswick

**Affiliations:** Musculoskeletal Research Unit, School of Clinical Sciences, Learning and Research Building (Level 1), Southmead Hospital, University of Bristol, Southmead Road, Bristol, BS10 5NB UK; North Bristol NHS Trust, Bristol, BS10 5NB UK; Royal Devon and Exeter NHS Foundation Trust, Exeter, EX2 7JU UK; Northern General Hospital, Sheffield Teaching Hospitals NHS Trust, Sheffield, S5 7AU UK; Oxford University Hospitals NHS Trust, Oxford, OX3 9DU UK; Cardiff and Vale University Health Board, Llandough, CF5 2LD UK; The Robert Jones and Agnes Hunt Orthopaedic Hospital NHS Foundation Trust, Oswestry, SY10 7AG UK; Wrightington, Wigan and Leigh NHS Foundation Trust, Wigan, WN6 9EP UK

**Keywords:** Infection, Hip replacement, Revision, One-stage, Two-stage, Individual participant data, Meta-analysis

## Abstract

**Background:**

Several aggregate published reviews have compared the effectiveness of one- and two-stage surgical revision to prevent re-infection following prosthetic hip infection and have reported inconsistent results. In addition, there were several features of these previous reviews which limited the validity of the findings. In the absence of a well-designed clinical trial, we propose the Global Infection Orthopaedic Management (INFORM) collaboration, a worldwide collaborative systematic review and meta-analysis of individual participant data (IPD) to address the existing uncertainties.

**Methods:**

Cohort studies (prospective or retrospective) and randomised controlled trials conducted in unselected patients with infection treated exclusively by one- or two-stage revision and reporting re-infection outcomes within 2 years of revision will be retrieved by searching the following databases: MEDLINE, EMBASE, Web of Science, Cochrane Database of Systematic Reviews, Cochrane Central Register of Controlled Trials and the WHO International Clinical Trials Registry Platform. Reference lists of relevant studies will be manually scanned and there will be email contact with investigators of grey literature and conference abstracts. Investigators will be invited to join the Global INFORM collaboration and share their individual level data. The primary outcome of the analyses will be incidence of re-infection within 2 years of commencement of revision surgery. Primary analyses will be conducted comparing the one-stage to the two-stage surgical revision. IPD analyses will be based on Cox proportional hazard (PH) models estimated for each study separately. Study-specific log hazard ratios will be combined using random-effects meta-analysis with fixed-effects meta-analysis in subsidiary analyses. Hazard ratios for re-infection according to different individual level characteristics such as sex, age groups, body mass index and comorbidities will also be assessed.

**Discussion:**

The analyses will enable a consistent approach to the definition of re-infection outcomes, more detailed analyses under a broader range of circumstances and exploration of potential sources of heterogeneity and produce much more valid and precise estimates of re-infection outcomes.

**Systematic review registration:**

PROSPERO 2015: CRD42015016664

**Electronic supplementary material:**

The online version of this article (doi:10.1186/s13643-015-0044-0) contains supplementary material, which is available to authorized users.

## Background

Hip replacement is widely used to treat pain associated with diseased or damaged joints. Deep prosthetic infection is an uncommon but serious complication of hip replacement with estimated incidence in the UK population of about 0.70% [1]. Prosthetic joint infection occurring within 2 years of hip replacement is mainly a consequence of the surgical intervention [2] and are commonly associated with extreme pain, restricted movement, feelings of isolation, insecurity and hopelessness and may even lead to death [3,4]. Treatment options for prosthetic hip infection include the following: surgical removal of dead, damaged and infected tissue (debridement) with prosthesis retention and long-term antibiotic treatment; debridement and the exchange of modular components; one-stage revision; two-stage revision; excision of the joint replacement; or amputation. One-stage surgical revision involves prosthesis removal, debridement, antibiotic treatment and joint replacement in the same surgical operation. In the two-stage revision, a temporary ‘spacer’ may be fitted, which is replaced in the second operation typically 2 to 6 months later. The two-stage revision is used commonly and has been traditionally regarded as more effective in treating infection [5,6]. In England and Wales, about 500 hip revision procedures per year following prosthetic joint infection are carried out, with 30% being one-stage revision, 64% being two-stage and 6% being excision [7]. Despite the opportunities for additional microbial strategies [8] with the two-stage procedure, patients who undergo this procedure require additional hospital admissions, undergo further major surgery and experience considerable pain and disability during the period between operations and sometimes after the revision [9]. A two-stage revision may also cost about 70% more than a one-stage revision [10]. There is an increasing interest in the use of the single-stage revision as it may be associated with significantly less morbidity and disability; may eliminate the requirement for prolonged stay in the hospital; and overall healthcare costs of this procedure may be less than the two-stage procedure. There is currently uncertainty regarding the best treatment option.

There have been no randomised controlled trials comparing one- and two-stage revision procedures. However, several observational studies have assessed re-infection outcomes following the one-stage or two-stage surgical revisions and have reported inconsistent results. Wolf and colleagues conducted a literature review in 2008 and used a decision analysis to compare the one- and two-stage revision strategies [11]. In their search of only MEDLINE and the American and British editions of *The Journal of Bone and Joint Surgery*, 11 two-stage (321 patients) and eight one-stage (576 patients) studies were included in the review. In pooled analysis, they reported an increased re-infection rate after one-stage (12.3%) compared to two-stage revision (6.5%) of infected total hip replacements [11]. There was however a higher mortality rate associated with the two-stage compared to one-stage. Using a Markov cohort simulation decision analysis, they reported that the overall balance of risk and benefit favours the one-stage approach in the treatment of infection after a total hip replacement. Lange and colleagues in their systematic review and meta-analysis included a total of 36 published studies (comprising 375 one-stage and 929 two-stage patients), identified from a search of PUBMED, EMBASE, Cochrane Library and the WHO International Clinical Trials Registry Platform databases till 2010 [12]. Re-infection rates of 13.1% and 10.4% were reported for one- and two-stage revision strategies, respectively. Our group has also recently assessed re-infection outcomes of one- and two-stage revision of infected hip replacements using published studies that included populations representative of patients in routine clinical practice [13]. In a search of EMBASE, MEDLINE and Cochrane databases, 11 one-stage (1,225 patients) and 28 two-stage (1,188 patients) studies were included in the review. Pooled random-effects meta-analysis yielded a lower re-infection rate at 2 years for one-stage-revision (8.6%) compared to a two-stage revision (10.2%), although this difference was not significant. The inconsistent evidence does not conclusively support a specific revision strategy for prosthetic hip infection. In addition, there were several features of these aggregate reviews which limit the validity of the findings. Firstly, none of the reviews performed a detailed quality assessment of the included studies. Secondly, the heterogeneous definition of re-infection outcomes by the studies included did not allow reliable comparison of the findings. Though our review attempted to minimise this by assessing only re-infection outcomes within 2 years of revision surgery, this approach was not possible in all studies. Thirdly, there was substantial heterogeneity amongst contributing studies which could not be adequately explored. Finally, majority of these reviews did not conduct any subgroup analysis or assess the possibility of publication bias.

Given these limitations of aggregate published data, the results of the previous reviews may be misleading and potentially promote inappropriate recommendations for a specific strategy for revision of infected hip prostheses. To compare the effectiveness of one-stage and two-stage revision will require robust evidence from a carefully designed randomised clinical trial. With the low incidence of prosthetic hip infection, an appropriate definitive randomised trial with re-infection as the primary outcome is currently unlikely. In the absence of such a clinical trial, we propose the Global Infection Orthopaedic Management (INFORM) collaboration, a worldwide collaborative systematic review and meta-analysis of individual participant data (IPD) to address the existing uncertainties in the previous reviews. This will enable a consistent approach to the definition of re-infection outcomes, more detailed analyses under a broader range of circumstances, exploration of potential sources of heterogeneity and produce much more valid and precise estimates of re-infection outcomes.

### Aims and objectives

The main aim of this worldwide collaborative systematic review and meta-analysis of IPD is to compare the effectiveness of one- and two-stage surgical revision to prevent re-infection following prosthetic hip infection. Specific objectives are as follows:

1. To compare characteristics of patients undergoing one-stage or two-stage revision surgery following prosthetic hip infection
2. To assess average time to re-infection following one-stage or two-stage revision surgery
3. To assess incidence of re-infection (primary outcome) for one-stage or two-stage revision surgery
4. To compare risk of re-infection for one-stage compared to two-stage revision surgery following adjustment for potential confounders
5. To compare risk of re-infection for one-stage compared to two-stage revision surgery in clinically relevant subgroups
6. To compare other outcome measures such as patient reported function, readmission, pain and death (when data permits).

## Methods

We will conduct this systematic review and IPD meta-analysis using a predefined protocol and in accordance with methods recommended by the IPD Meta-analysis Methods Group of the Cochrane Collaboration [14] and other published guidelines [15]. Results will be reported in accordance with relevant aspects of PRISMA and MOOSE guidelines [16,17]. In accordance with the PRISMA 2015 statement [18,19], in the event of an important protocol amendment - the date of the amendment, description of the amendment and rationale for amendment will be provided. A revised protocol will be generated which will list the specific amendments made to the previous version. The lead investigator will be responsible for approving and supervising the documentation and implementation of the amendments.

### Data sources and search strategy

**Figure 1:**
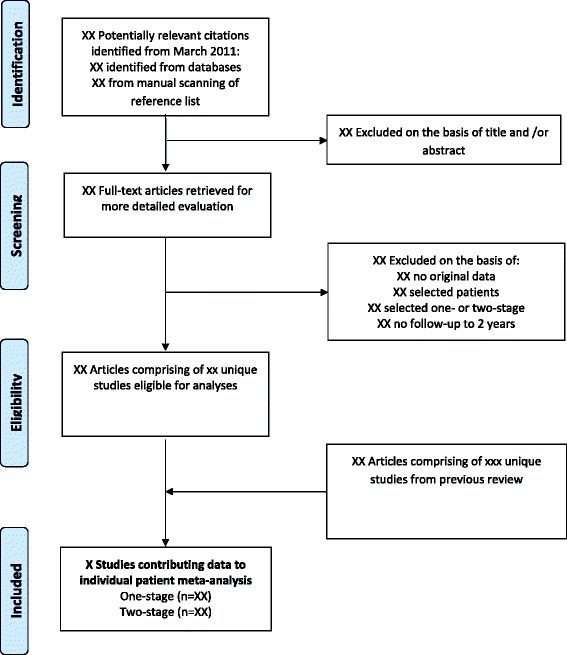
Draft PRISMA flow diagram.

We will update the literature search to identify new studies published since the completion of our systematic review [13] which compared the effectiveness of one-stage and two-stage revision for re-infection outcomes following prosthetic hip infection. We will systematically search for longitudinal studies (retrospective, prospective or randomised controlled trials) reporting re-infection outcomes following one- or two-stage surgical revision of infected hip prosthesis in MEDLINE, EMBASE, Web of Science, Cochrane Database of Systematic Reviews, Cochrane Central Register of Controlled Trials and the WHO International Clinical Trials Registry Platform from March 2011 (date of our last search for the previous review) to date. The computer-based searches will combine free and MeSH search terms and combination of keywords related to hip replacement, infection and revision with focus on one- and two stage revision surgeries. There will be no restrictions on language. Reference lists of retrieved articles will be manually scanned for all relevant additional studies and review articles. An additional Microsoft Word file shows the search strategy in more detail [see Additional file 1]. To minimise publication bias, information on studies in progress (Clinical Trials.gov) and studies reported in the grey literature [Systems for Information in Grey Literature (SIGLE) and Dissertation abstracts] will be sought. See Figure 1 for preliminary PRISMA flow of studies.

### Eligibility criteria

Studies will be included if they involve unselected patients with prosthetic hip infection (over 18 years of age) treated exclusively by one-stage or two-stage revision and had at least been followed up for 2 years for re-infection outcomes after revision surgery. Studies that report case series of methods in selected group of patients (such as subsamples of patients who received revision in one or two stages or patients with a specific infection), did not include patients with less than 2 years of follow-up, and studies with less than ten participants will be excluded from the review.

### Study selection and quality assessment

Two investigators will independently review titles and abstracts for eligibility. If either reviewer determines that a study may be eligible based on title or abstract review, then a full-text article review will be completed. Each article will be assessed using the inclusion criteria above and any disagreement regarding eligibility of an article will be discussed, and agreement reached by consensus with a third reviewer. All studies in languages other than English will be translated into English. Methodological quality will be assessed based on the Methodological Index for Non-Randomised Studies (MINORS), a validated instrument which is designed for assessment of methodological quality of non-randomised studies in surgery [20]. For non-comparative studies, it uses eight pre-defined domains namely: a clearly stated aim, inclusion of consecutive patients, prospective collection of data, endpoints appropriate to the aim of the study, unbiased assessment of the study endpoint, follow-up period appropriate to the aim of the study, loss to follow-up less than 5% and prospective calculation of the study size. For each item, MINORS assigns 0 for not reported, 1 for reported but inadequate, or 2 for reported and adequate. The global ideal score is 16.

### Establishment of the Global INFORM collaboration

Investigators of eligible studies will be invited to join the collaboration by providing us with individual level data. We will identify contact information from the published studies or an online search. Each principal investigator will be contacted and provided with the protocol and a cover letter. Based on our recent published review [13], we have identified key studies that may potentially contribute data to this collaboration. We are also in the process of updating our previous review as several important studies have been published since its publication. A number of authors of studies have expressed initial interest to collaborate in this effort [21-28] (Table 1).

**Table 1:** **Characteristics of studies with provisional support to share individual level data**

**Lead author, publication date (reference number)**	**Location**	**Year of study**	**Mean/median age (years)**	**% male**	**Follow-up mean/median (months)**	**Type of re-implantation**	**Use of spacer**	**Number of re-infections**	**Number of participants**	**Type of revision**
Elson *et al*. 1993 [21]	UK	NS	NS	NS	NS	NS	NA	33	235	One-stage
Whittaker *et al*. 2009 [22]	UK	1998 to 2003	69.0	49.0	49.0	Both	Spacer	6	43	Two-stage
Cabrita *et al*. 2007 [23]	Brazil	1996 to 2003	54.6	58.0	48.0	NS	Spacer	4	38	Two-stage
Ritter *et al*. 2010 [25]	USA	1969 to 2004	66.2	52.0	82.8	NS	NS	5	17	Two-stage
De Man *et al*. 2011 [24]	Switzerland	1985 to 2004	70.0	57.0	45.6	NS	NS	1	55	One-stage
Neumann *et al*. 2012 [26]	Austria	2000 to 2008	25 to 84^a^	56.8	67.0	Cementless	Spacer	1	44	Two-stage
Schwarzkopf *et al*. 2014 [27]	USA	2001 to 2011	62.3	46.9	32.4	Cemented	Spacer	3	62	Two-stage
Zeller *et al*. 2014 [28]	France	2002 to 2010	71.0	58.0	41.6	Cementless	NA	6	157	One-stage

NA, not applicable; NS, not stated; Both, some participants had cemented re-implantation and others cementless.

^a^Age range.

### Data collection

Investigators will be provided with a list of relevant study variables that could be used in the analyses (Table 2), and data dictionaries will be requested. We will accept data in all formats but preferably in STATA, SPSS or Microsoft Excel. Individual level data collected will be cleaned, coded and entered into a single STATA database (Stata Corp, College Station, TX, USA).

**Table 2:** **Study variables to be requested for individual participant data meta-analysis**

**Broad classification**	**Variables**
Socio-demographic characteristics	Country in which study was carried out, age, sex, body mass index (weight and height) and smoking status
Past medical/surgical history	Previous hip surgery, other joint surgery and comorbidities (for example, history of diabetes), Charnley classification, Charlson index, ASA grade
Infection characteristics before revision surgery	Duration between index implantation and occurrence/diagnosis of infection, duration between diagnosis of infection and revision surgery
Baseline laboratory data for infection	CRP, ESR, leucocytes, causative organism, neutrophil count, white cell count, synovial fluid white cell count, IL-6
Characteristics of surgical revision	Date of revision, type of re-implantation, type of fixation (cemented or uncemented), use of spacer, type of spacer (static or articulating), use of antibiotics in cement or spacer, time interval between stages for two-stage procedure, diagnosis of re-infection, date of diagnosis of re-infection or date of last follow-up for participants without re-infection, time to re-infection or last follow-up (days or years), antibiotics used and duration of antibiotics
Intervention	One- or two-stage revision

ASA, American Society of Anaesthesiologists; CRP, C-reactive protein; ESR, erythrocyte sedimentation rate; IL-6, interleukin-6.

### Statistical analysis

Re-infection within 2 years of hip revision surgery will be used as the primary outcome. Secondary outcomes will include further treatments (such as re-revision surgery), pain, functional outcomes and death. No formal sample size requirements are necessary for the meta-analysis. Primary analyses will be conducted comparing the one-stage to the two-stage (reference category) procedure. IPD analyses will be based on Cox proportional hazard (PH) models estimated for each study separately. All PH models will be stratified by sex and adjusted for age, body mass index, comorbidities and other potential confounders. Study-specific log hazard ratios (HRs) will be combined using random-effects meta-analysis. Between-study heterogeneity will be quantified by the I^2^ statistic. Supplementary analyses will combine log HRs using fixed-effects meta-analysis. Given the distortions (for example, non-convergence of statistical models) that can appear in analyses involving small strata (such as studies contributing less than ten outcomes to a specific analysis) when using two-stage PH models, a single-stage PH model which maintains clustering of the data will also be considered. In subsidiary analyses, a propensity score approach will also be used to compare re-infection outcomes between the two types of revisions. Comparison of the one- to two-stage approach for re-infection incidence may vary under different circumstances. There is therefore no reason to expect *a priori* that the results will be similar for example in patients with/without a history of diabetes or in regions that have different underlying infection patterns. It is therefore important to investigate the comparisons according to different level characteristics. Therefore, primary analyses will be repeated to calculate HRs for re-infection according to different individual and study level characteristics such as sex, age groups, body mass index, comorbidities, major geographical regions (that is, Europe/North America/Australia/NZ, South Asia and East Asia) and study quality (low *versus* high quality studies). Sensitivity analysis will include determining HRs for re-infection on exclusion of low-quality studies. The potential impact of publication bias and unavailable data will be explored in accordance with methods proposed by Ahmed and colleagues [29]. Funnel plots will also be constructed to examine the likelihood of publication bias. All analyses will be conducted using Stata version 13 (Stata Corp, College Station, TX, USA).

### Ethical issues

We will not seek ethical approval for this systematic and IPD meta-analysis, because we will be collecting and synthesising the same data from previously published studies in which informed consent and ethical approval has already been obtained by the study investigators (which will be confirmed in the published study and in writing). Secondly, our systematic review and IPD meta-analysis will be addressing similar questions to the research question for which the data were collected. Thirdly, we will request investigators to submit anonymised datasets. Finally, our Research Ethics Committee has confirmed that ethical approval is not required for the present study, given the above reasons. Our research is supported by an established patient forum [30], and we will seek advice on issues relating to review conduct and dissemination of results.

### Proposed collaborative publication arrangements

It is proposed that the following collaborative arrangements should be used in this effort: 1) scientific papers will be published in the name of a collaborative group (for example, ‘Global Infection Orthopaedic Management Collaboration’), 2) the members of a writing committee are to be listed at the end of the paper with a small number of representatives (for example, three from each of the contributing studies sharing data), 3) the sequence of names in the writing committee is to be arranged to help demonstrate the collaborative nature of this effort (for example, each study will be able to include one ‘starred’ joint first author and one ‘starred’ joint final author), 4) in addition to the researchers listed in the writing committee and the analytical subcommittee, it may be possible to list under a separate heading additional scientists who have made contributions to this effort (for example, under the heading ‘Investigators’, it may be possible to list principal investigators from each of the component studies, subject to the approval of the paper’s target journal, which has not yet been determined). The Cochrane IPD meta-analysis method group recommends a collaborators’ meeting for preliminary presentation of meta-analyses [31]. We anticipate that this will take the form of a teleconference but will explore the feasibility of holding a workshop locally or at an appropriate international conference.

## Discussion

The present analysis will differ from the previous reviews in the following main ways: (i) access to individual level data should enable a consistent approach to the definition of the primary outcome (2-year incidence of re-infection), a common approach across studies to statistical analyses and a consistent approach to adjustment for potential confounders, a greater ability to explore and identify sources of between-study heterogeneity, comparison of the one- to two-stage revision for re-infection incidence under different level characteristics and ability to assess secondary outcomes such as patient-reported function, pain and death; (ii) statistical power - as the present analysis will involve several-fold more participants, it should be substantially more powerful and precise than previous individual studies and reviews; (iii) generalisability - as it is anticipated to include studies with populations representative of patients in routine clinical practice, the results from the present meta-analysis should be more generalisable. Moreover, inclusion in the proposed analysis of key prospective studies worldwide should help avoid biases due to selective inclusion of studies and enhance the generalisability of the study results.

### Registration

The protocol for the systematic review and IPD meta-analysis has been registered with PROSPERO, the international prospective register of systematic reviews, (PROSPERO 2015: CRD42015016664). Available from http://www.crd.york.ac.uk/PROSPERO/display_record.asp?ID=CRD42015016664.

## Additional file

Additional file 1:
**Search strategy for MEDLINE.**

## Abbreviations

HR: hazard ratio
INFORM: Infection Orthopaedic Management
IPD: individual participant data
MINORS: Methodological Index for Non-Randomised Studies
PH: proportional hazards
PRISMA: Preferred Reporting Items for Systematic Reviews and Meta-Analyses
