# Supplementary material for: Re-infection outcomes following one- and two-stage surgical revision of infected hip prosthesis in unselected patients: protocol for a systematic review and an individual participant data meta-analysis
Source: Syst Rev. 2015 Apr 25;4:58. doi: 10.1186/s13643-015-0044-0 (PMC4424893; doi:10.1186/s13643-015-0044-0)
Supplement: Additional file 1: — Search strategy for MEDLINE. [file 13643_2015_44_MOESM1_ESM.doc]

**Additional file 1:** Search strategy for MEDLINE

--------------------------------------------------------------------------------

1 exp Prosthesis-Related Infections/ or prosthesis-related infection*.mp. or exp Sepsis/ (101971)

2 exp Infection/ or exp Wound Infection/ or exp Surgical Wound Infection/ or infection*.mp. (1603015)

3 wound infection.mp. or exp Wound Infection/ (44687)

4 arthroplasty.mp. or exp Arthroplasty, Replacement/ or exp Arthroplasty/ or exp Arthroplasty, Replacement, Hip/ (52003)

5 exp Arthroplasty, Replacement, Hip/ or exp Arthroplasty, Replacement/ or Replacement.mp. (204405)

6 exp Hip/ or exp Arthroplasty, Replacement, Hip/ or hip.mp. (107123)

7 exp Hip Prosthesis/ or exp Arthroplasty, Replacement, Hip/ or hip replacement.mp. (31655)

8 exp Hip Prosthesis/ or total hip.mp. or exp Arthroplasty, Replacement, Hip/ (34732)

9 hip arthroplasty.mp. (12045)

10 total hip replacement.mp. or exp Arthroplasty, Replacement, Hip/ (20706)

11 exp Arthroplasty, Replacement, Hip/ or total hip arthroplasty.mp. (20637)

12 exp Arthroplasty, Replacement, Hip/ or exp Hip Prosthesis/ or hip prosthes*.mp. (30397)

13 1-stage.mp. (1576)

14 2-stage.mp. (2828)

15 one stage.mp. (8705)

16 two stage.mp. (15977)

17 one-stage.mp. (8705)

18 two-stage.mp. (15977)

19 single stage.mp. (4349)

20 single-stage.mp. (4349)

21 prosthesis exchange.mp. (15)

22 direct exchange.mp. (153)

23 direct-exchange.mp. (153)

24 Arthroplasty, Replacement, Hip/ or revision arthroplasty.mp. (18017)

25 exp Arthroplasty, Replacement, Hip/ or staged revision.mp. (17490)

26 reoperation.mp. or exp Reoperation/ (77278)

27 reimplantation.mp. or exp Replantation/ (10449)

28 1 or 2 or 3 (1606087)

29 4 or 5 or 6 or 7 or 8 or 9 or 10 or 11 or 12 (295528)

30 13 or 14 or 15 or 16 or 17 or 18 or 19 or 20 or 21 or 22 or 23 or 24 or 25 or 26 or 27 (129343)

31 28 and 29 and 30 (5795)

32 limit 31 to yr="2011 -Current" (1602)

***************************

Each part was specifically translated for searching the other databases (EMBASE, Web of Science, and Cochrane databases)
